# 2-(Maleimidomethyl)-1,3-Dioxanes (MD): a Serum-Stable Self-hydrolysable Hydrophilic Alternative to Classical Maleimide Conjugation

**DOI:** 10.1038/srep30835

**Published:** 2016-08-09

**Authors:** Igor Dovgan, Sergii Kolodych, Oleksandr Koniev, Alain Wagner

**Affiliations:** 1 Laboratory of Functional ChemoSystems (UMR 7199), LabEx Medalis, University of Strasbourg, France; 2 Syndivia SAS, 650 Boulevard Gonthier d’Andernach, 67400 Illkirch, France

## Abstract

The vast majority of antibody-drug conjugates (ADC) are prepared through amine-to-thiol conjugation. To date, *N*-Succinimidyl-4-(maleimidomethyl) cyclohexanecarboxylate (SMCC) has been one of the most frequently applied reagents for the preparation of ADC and other functional conjugates. However, SMCC-based conjugates suffer from limited stability in blood circulation and from a hydrophobic character of the linker, which may give rise to major pharmacokinetic implications. To address this issue, we have developed a heterobifunctional analogue of a SMCC reagent, i.e., sodium 4-(maleimidomethyl)-1,3-dioxane-5-carbonyl)oxy)-2,3,5,6- tetrafluorobenzenesulfonate (MDTF) for amine-to-thiol conjugation. By replacing the cyclohexyl ring in the SMCC structure with the 1,3-dioxane, we increased the hydrophilicity of the linker. A FRET probe based on MD linker was prepared and showed superior stability compared to the MCC linker in human plasma, as well as in a variety of aqueous buffers. A detailed investigation demonstrated an accelerated succinimide ring opening for MD linker, resulting in stabilized conjugates. Finally, the MDTF reagent was applied for the preparation of serum stable antibody-dye conjugate.

*N*-Succinimidyl 4-(maleimidomethyl)cyclohexanecarboxylate (SMCC) is one of the most used reagents in bioconjugation^1^,^2^. This heterobifunctional reagent contains an *N*-Succinimidyl (NHS) ester that reacts with amines, yielding a peptide bond and a maleimide group that reacts with thiols, resulting in the formation of a thioester. Both groups are joined together by a cyclohexyl ring, which has been shown to increase the aqueous stability of the maleimide group^3^. Due to the high abundance of both amines (e.g., lysine residues) and thiols (e.g., cysteine residues) in biological molecules, the SMCC reagent has become an indispensable tool for the modification of biomolecules.

The applications of SMCC include preparation of hapten-carrier conjugates^4^,^5^, antibody-enzyme conjugates^6^,^7^,^8^, immunotoxins^9^ and perhaps the most advanced application to date, generation of antibody-drug conjugates (ADC)^10^,^11^. Indeed, one of the two marketed ADCs, trastuzumab emtansine^12^, as well as other mertansine-based ADC in clinical development, are prepared via SMCC-mediated conjugation^10^,^11^,^13^, in which a highly potent drug is directly linked to an antibody through the MCC linker.

Despite its high applicability, some issues arise from the relatively hydrophobic character of SMCC. Precipitation of the linker in aqueous media, as well as aggregation and precipitation of resulting bioconjugates may occur, decreasing both conjugation efficiency and yield. This issue is of particular importance for the development of mertansine-based ADCs, where the drug is connected to an antibody through the MCC linker, without additional cleavable peptides or other elements that can increase water solubility.

To address the issue of reagent precipitation, a sulfo-SMCC linker containing a sulfonate group on the NHS ring was developed^14^. However, the linker structure remained unchanged and thus, the problem pertaining to linker innate hydrophobicity (causing aggregation and precipitation of bioconjugates) remained unsolved.

## Results and Discussion

**Figure 1:**
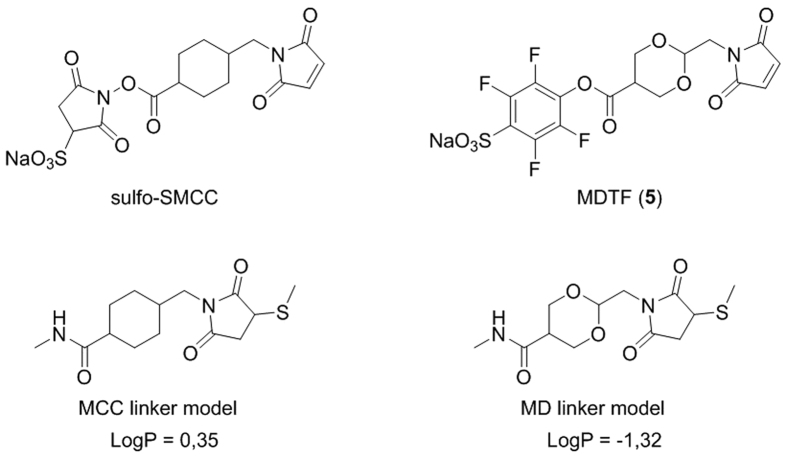
SMCC and MDTF reagents, the resulting linker models and their calculated LogP values. LogP values indicate higher hydrophilicity of the MD linker model.

In an effort to address this issue, we designed a new SMCC-like reagent 5 with increased hydrophilicity of the linker core structure. This was achieved by substitution of the cyclohexyl ring by the 1,3-dioxane analogue (Fig. 1). By fitting two oxygen atoms into the structure, the calculated LogP value of the linker decreased by 1.67 units. Moreover, we replaced the sulfo-NHS-activated ester with the 4-sulfotetrafluorophenylester in order to increase the solubility of the final product in water, which is an important parameter for biological applications^15^.

**Figure 2:**
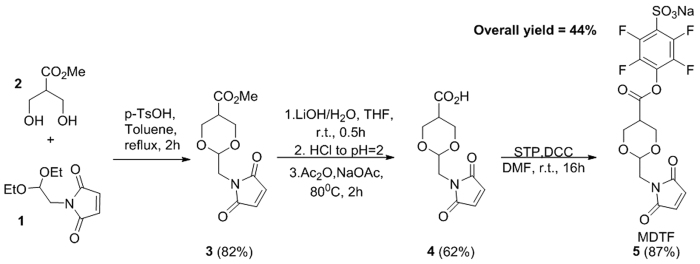
Synthesis of MDTF reagent.

We developed a new heterobifunctional reagent, the sodium 4-(maleimidomethyl)-1,3-dioxane-5-carbonyl)oxy)-2,3,5,6-tetrafluorobenzenesulfonate **5** (MDTF) in three steps from readily available precursors 1 and 2 (Fig. 2). First, the reaction between 1 and 2 was carried out by refluxing their mixture in toluene, in the presence of a catalytic amount of *p*-TsOH in order to give 1,3-dioxane 3 in 82% yield. Then, hydrolysis of 3 with lithium hydroxide solution (THF/water) led to simultaneous de-esterification of carboxyl function and maleimide ring opening. The latter was then transformed to 4 (cis-isomer) using previously reported reaction conditions^3^ in 62% yield. Finally, the activation of the carboxylic function of 4 with sodium salt of 4-sulfo-2,3,5,6-tetrafluorophenol (STP) gave the targeted activated ester 5 in 44% overall yield. Reactions were reproduced three times on a scale ranging from hundreds of milligrams to several grams.

**Figure 3:**
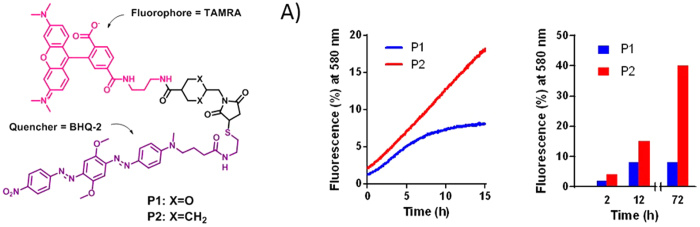
Structures of FRET-based probes P1 and P2 synthesized by amine-to-thiol coupling using MDTF and sulfo-SMCC reagents with 1 eq. of TAMRA-NH_2_ and 1 eq. of BHQ-2-SH. (**A**) Side-by-side comparison of the stability of probes P1 and P2 (1 μM) in human plasma at 37 °C. Fluorescence of probes P1 and P2 was monitored at 580 nm and was normalized to the fluorescence of a solution of TAMRA-NH_2_ (1 μM) and BHQ-2-SH (1 μM) in human plasma.

In order to assess the stability of the linker in biological media and at different pH we synthesized two FRET-based probes **P1** and **P2** using MDTF and sulfo-SMCC reagents respectively through amine-to-thiol conjugation of 1 eq. of fluorophore-amine (TAMRA-NH_2_) and 1 eq. of quencher-thiol (BHQ-2-SH). The probes were purified using semi-preparative HPLC in order to remove all traces of the starting materials (Fig. 3). These probes were not fluorescent, as the quencher and the fluorophore were linked together through MD- or MCC-linker, but cleavage of the linker or substitution of BHQ-2-SH by other thiol-containing molecules such as human serum albumin (HSA) resulted in the appearance of the fluorescence signal.

To test the stability of the linkers we incubated probes **P1** and **P2** (1 μM) in different buffers (TRIS, PBS) at various pH (5.5 ÷ 9.0), as well as in human plasma and in 1 M HCl at 37 °C (see supporting information). The appearance of fluorescence was monitored at 580 nm over 15 h and normalized using a solution of TAMRA-NH_2_ (1 μM) and BHQ-2-SH (1 μM) in appropriate media as a positive control.

Interestingly, despite the presence of an acetal function in its structure, the MD linker appeared to be more stable than MCC, even at pH = 0 (see supporting information). We also found that the fluorescence observed during incubation of **P1** in human plasma reached a plateau after 12 hours (Fig. 3), while **P2** exhibited linearly increasing fluorescence. The latter was demonstrated as being the result of a gradual exchange of BHQ-2-SH by the thiol of human serum albumin (HSA) present in human plasma^16^. This linear fluorescence increase in P2 was maintained and after 72 hours provided 40% of linker cleavage. In contrast, the fluorescence of P1 remained unchanged after reaching a plateau.

**Figure 4:**
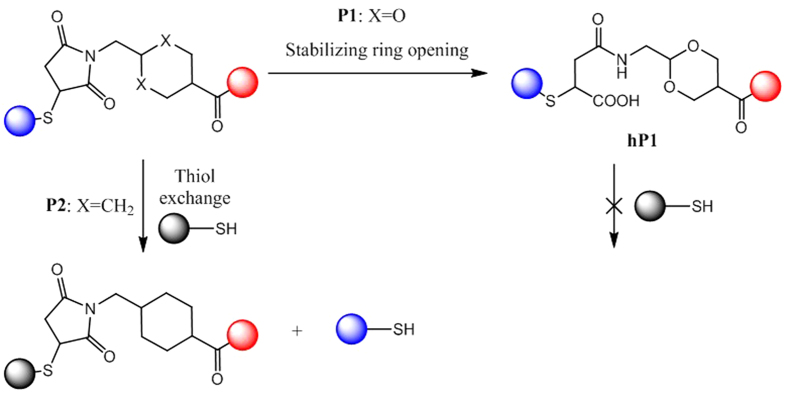
The high rate of irreversible hydrolysis for the succinimide motif for P1 led to succinamic acid hP1, which does not undergo thiol exchange reaction. In the case of P2, thiol exchange occurred faster than the stabilizing succinimide hydrolysis.

We hypothesized that the difference in behavior of similar scaffolds was due to the hydrolysis of the succinimide motif in the case of **P1**, which led to the succinamic acid **hP1**, which is known to be stable toward thiol exchange (Fig. 4).

To confirm this hypothesis, we measured the succinimide hydrolysis rates in human plasma of **P1** and **P2** via LC-MS analysis. Hence, probes **P1** and **P2** (50 μL each) were incubated in human plasma containing 10% of DMSO at 37 °C. Aliquots were analyzed by HPLC after the precipitation of proteins by the addition of acetonitrile.

**Figure 5:**
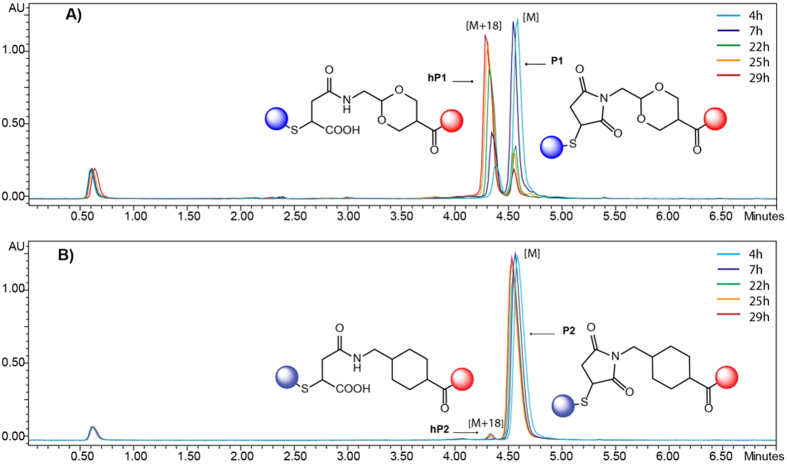
Incubation of FRET probes P1 and P2(50 μM) in human plasma at 37 °C and subsequent analysis of mixture composition by LC-MS analysis at 550 nm. (**A**) Hydrolysis of succinimide of the MD-based probe **P1** yielding succinamic acid **hP1**. (**B**) Only trace amount of hydrolysed product was observed for the MCC-based probe **P2**.

As expected, a peak corresponding to succinamic acid **hP1** was observed for the probe **P1** and the reaction was almost complete after 29 hours, while for the probe **P2**, only a trace amount of hydrolyzed product **hP2** could be detected after 29 hours (Fig. 5).

MD-based linkers therefore appear to offer an interesting possibility for self-stabilization of the resulting conjugates via a succinimide ring opening. It is worth noting that it has earlier been reported that stabilization of maleimide-thiol conjugates, achieved by hydrolysis of the succinimide ring, can be induced by modulation of the site of conjugation to an antibody^17^,^18^ by an amino group adjacent to the maleimide^19^, by electron withdrawing *N*-substituents^20^,^21^ or by using *N*-aryl maleimides^22^. In most cases, buffers with high pH values are required to achieve hydrolysis^20^,^21^. Alternatively, to enable access to serum stable conjugates, maleimide can be replaced by other thiol-reactive groups such as 3-arylpropionitrile^23^,^24^ or phenyloxadiazole sulfones^25^; alternatively, bioorthogonal methods of conjugation can be applied^26^.

**Figure 6:**
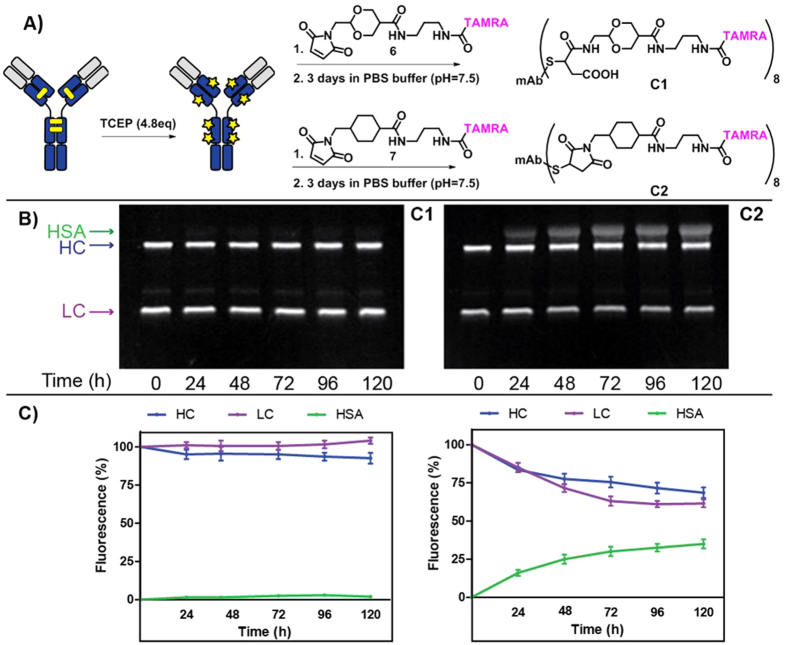
(**A**) Preparation of Trastuzumab-TAMRA conjugates C1 and C2 with a dye-to-antibody ratio (DAR) = 8 via the maleimide-thiol reaction of reduced Trastuzumab with MD-TAMRA 6 and MCC-TAMRA 7, followed by the mild hydrolysis of succinimide in a PBS buffer (10 mM, pH = 7.5) at 37 °C (see supporting information). (**B**) Fluorescent SDS-PAGE analysis (excitation at 525 nm) of MD- and MCC-based antibody-dye conjugates after incubation in human plasma. Two lanes correspond to the labeled heavy (HC) and light chains (LC) of the antibody, the third line corresponds to the labeled human serum albumin (HSA) formed via thiol exchange reaction. (**C**) Quantitative analysis of conjugate stability in human plasma demonstrated 38% of payload transfer to HSA over 120 hours for the MCC-based conjugate C2 in contrast to 3% for the MD-based conjugate C1.

Encouraged by these results, we then decided to test the MDTF reagent for the preparation of an antibody-fluorophore (TAMRA) conjugate and to evaluate whether self-hydrolysis properties can be used to prepare stable conjugates. To this end, a side-by-side comparison with a sulfo-SMCC reagent was carried out (Fig. 6).

First, a reaction between MDTF or sulfo-SMCC and TAMRA-NH_2_ using classical conjugation conditions was conducted to yield payloads **6** and **7**, respectively (see supporting information). Then, a complete reduction of the interchain disulfide bonds of Trastuzumab was performed by incubation with TCEP (4.8 equiv.) at 37 °C for 2 hours. The reduced antibody was then reacted with **6** or **7** at 4 °C for 1 hour to yield the corresponding conjugates as 5 mg/mL solutions in a PBS buffer (10 mM, pH = 7.5) after purification by gel filtration chromatography. The ESI-MS analysis^27^ confirmed the dye-to-antibody ratio value of 8 for both conjugates **C1** and **C2** (Fig. 6).To trigger succinimidyl hydrolysis, as well as conjugates **C1** and **C2** were maintained in the PBS buffer used for conjugation (10 mM, pH = 7.5) at 37 °С for 70 hours. Following stabilization, both conjugates were incubated in human plasma for five days. Aliquots were taken every 24 hours and analyzed by SDS PAGE. In addition to the two lanes corresponding to the labeled heavy and (HC) light chains (LC) of the antibody, the MCC-based conjugate showed the gradual appearance of a third lane, corresponding to the transfer of the fluorophore to the HSA^16^. Quantitative analysis via integration of the fluorescent signal of this lane showed 38% degradation over 120 hours for MCC-based conjugates. In contrast, the MD-based conjugate **C1** did not show any noticeable transfer of payload to HSA and only 3% degradation was observed after 120 hours of incubation.

In summary, we have developed a new heterobifunctional reagent (MDTF) for amine-to-thiol conjugation that indicates similar reactivity towards sulfhydryl-containing molecules as the SMCC. Substitution of a cyclohexyl ring by a dioxine ring increased hydrophilic character in the new MD linker compared to the classical MCC linker. Interestingly, the MD linker underwent self-stabilization in mild conditions via a succinimide ring opening. The resulting succinamic acid-containing linker is not prone to the undesirable thiol exchange reaction. In fact, a MD-based FRET probe incubated in human plasma showed a considerably higher self-stabilization rate compared to the MCC-based probe. This hydrolytic stabilization process was shown to be efficient for the preparation of serum stable antibody conjugates.

## Additional Information

**How to cite this article**: Dovgan, I. *et al*. 2-(Maleimidomethyl)-1,3-Dioxanes (MD): a Serum-Stable Self-hydrolysable Hydrophilic Alternative to Classical Maleimide Conjugation. *Sci. Rep.* **6**, 30835; doi: 10.1038/srep30835 (2016).

## Supplementary Material

Supplementary Information
