# Supplementary material for: 2-(Maleimidomethyl)-1,3-Dioxanes (MD): a Serum-Stable Self-hydrolysable Hydrophilic Alternative to Classical Maleimide Conjugation
Source: Sci Rep. 2016 Aug 9;6:30835. doi: 10.1038/srep30835 (PMC4977557; doi:10.1038/srep30835)
Supplement: Supplementary Information [file srep30835-s1.pdf]

Supporting information

2-(Maleimidomethyl)-1,3-Dioxanes (MD): a Serum-Stable Self-hydrolysable Hydrophilic Alternative to Classical Maleimide Conjugation

Igor Dovgan, Sergii Kolodych, Oleksandr Koniev and Alain Wagner *

Laboratory of Functuional ChemoSystems

LaBex MEDALIS, UMR 7199 F

Faculté de Pharmacie

Université de Strasbourg

74 route du Rhin

CS 60024

67401 ILLKIRCH CEDEX

Email: alwag@unistra.fr

General Methods

General experimental procedures: Unless otherwise indicated, reactions were carried out under an atmosphere of argon in flame-dried glassware with magnetic stirring. Air and/or moisture-sensitive liquids were transferred via syringe. When required, solutions were degassed by bubbling of argon through a needle. Organic solutions were concentrated by rotary evaporation at 25-60 °C at 15-30 torr. Analytical thin layer chromatography (TLC) was performed using plates cut from glass sheets (silica gel 60F-254 from Merck). Visualization was achieved under a 254 or 365 nm UV light and by immersion in an appropriate revelation solution. Column chromatography was carried out as “Flash Chromatography” using silica gel G-25 (40-63 µm) from Macherey-Nagel.

Materials: All reagents were obtained from commercial sources and used without prior purifications. Dry solvents were obtained from Sigma-Aldrich. Instrumentation: 1H and 13C NMR spectra were recorded at 23°C on Bruker 400 and 500 spectrometers. Recorded shifts are reported in parts per million (δ) and calibrated using residual non-deuterated solvent. Data are represented as follows: chemical shift, multiplicity (s = singlet, d = doublet, t = triplet, q = quartet, quint = quintet, m = multiplet, br = broad), coupling constant (J, Hz) and integration. High resolution mass spectra (HRMS) were obtained using a Agilent Q-TOF (time of flight) 6520 and low resolution mass spectra using a Agilent MSD 1200 SL (ESI/APCI) with a Agilent HPLC1200 SL. Antibody MS experiments were performed on an electrospray time-of-flight mass spectrometer MS (LCT, Waters, Manchester) coupled to an automated chip-based nanoelectrospray device (Triversa Nanomate, Advion Biosciences, Ithaca, U.S.A.) operating in the positive ion mode. The LogP values were calculated using algorithms from fragment-based methods developed by the Medicinal Chemistry Project and BioByte (ChemOffice 2015).

Synthesis of MDTF

1,3-dimethyl 2,2-bis(hydroxymethyl)propanedioate

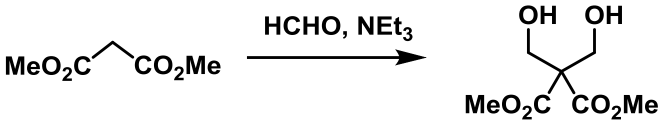

1,3-dimethyl 2,2-bis(hydroxymethyl)propanedioate was synthesised according to the literature procedure.[1]

5,5-dimethyl 2,2-dimethyl-1,3-dioxane-5,5-dicarboxylate

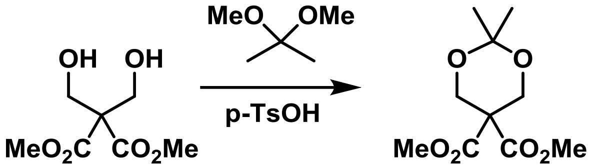

To a solution of 1,3-dimethyl 2,2-bis(hydroxymethyl)propanedioate (1 eq., 20 g, 104 mmol) in 2,2-dimethoxypropane (90.3 mL) was added TsOH·H_2O (10 %, 1.98 g, 10.4 mmol) at 25 °C. After the reaction mixture was maintained at room temperature for 1 h and was poured into a 5% (w/v) aqueous solution of $NaHCO_3$ (70 mL) and toluene (100 mL). The resulting organic layer was washed with brine (100 mL) and evaporated to afford the desired 5,5-dimethyl 2,2-dimethyl-1,3-dioxane-5,5-dicarboxylate (22.7 g, 97.7 mmol, 94 %) as a transparent oil, which was used in the next step without purification.

^{1}H NMR (400MHz, $CDCl_3$, δ ppm): 4.31 (s, 4 H), 3.79 (s, 6 H), 1.43 (s, 6 H).

^{13}C NMR (100MHz, $CDCl_3$, δ ppm): 168.4, 98.6, 62.5, 53.8, 53.0, 23.5.

MS(ESI) *m/z*: 232.09 $[M+H]^+$.

methyl 2,2-dimethyl-1,3-dioxane-5-carboxylate

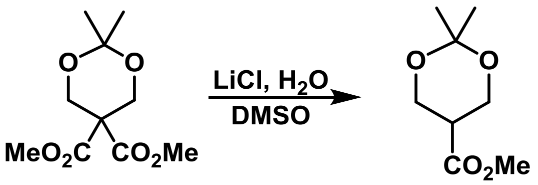

To a solution of 5,5-dimethyl 2,2-dimethyl-1,3-dioxane-5,5-dicarboxylate (1 eq., 21.4 g, 92.1 mmol) in DMSO (34 mL) were added LiCl (2 eq., 7.81 g, 184 mmol) and H_2O (1 eq., 1.66 g, 1.66 mL, 92.1 mmol). The reaction mixture was heated to 160 °C and stirred for 2 h, then cooled to 0 °C. To the reaction mixture were added water (120 mL) and EtOAc (240 mL), and the resulting mixture

was filtered. To the filtrate was added EtOAc (40 mL), the organic layer was washed with brine (40mL) and concentrated carefully under reduced pressure (the product is volatile) to afford methyl 2,2-dimethyl-1,3-dioxane-5-carboxylate (10.8 g, 61.7 mmol, 67%), which was used in the next step without purification.

^{1}H NMR (400MHz, $CDCl_3$, δ ppm): 4.02 - 4.12 (m, 4 H), 3.72 (s, 3 H), 2.78 - 2.86 (m, 1 H), 1.42 (s, 3 H), 1.45 ppm (s, 3 H) according to the literature[2].

methyl 3-hydroxy-2-(hydroxymethyl)propanoate (2)

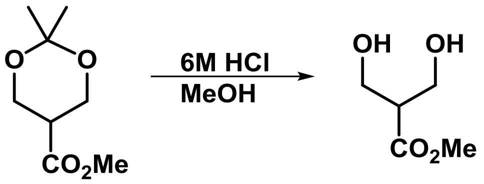

To a solution of methyl 2,2-dimethyl-1,3-dioxane-5-carboxylate (1 eq., 5.8 g, 33.3 mmol) in MeOH (42.1 mL) was added 0.086 mL of 6M HCl (0.56 eq., 1.84 g, 1.53 mL, 18.6 mmol) at 25 °C. The mixture was stirred for 12 h at 25 °C, and then $NaHCO_3$ (6.48 eq., 18.1 g, 215 mmol) was added. The reaction mixture was filtered and washed with EtOAc (50 mL) twice. The filtrate was concentrated *in vacuo* and the residue was purified by flash chromatography (EtOAc/Cyclohexane gradient: 0/100 to 100/0, then DCM 100%) to give methyl 3-hydroxy-2-(hydroxymethyl)propanoate (4.26 g, 31.6 mmol, 95 %) as a yellow oil.
^{1}H NMR (400MHz, $CDCl_3$, δ ppm): 3.99 (dd, J=12.2, 4.1 Hz, 4 H), 3.77 (s, 3 H), 2.74 (t, J=4.9 Hz, 1 H), 2.63 (br. s., 2 H).
^{13}C NMR (100MHz, $CDCl_3$, δ ppm): 173.7, 61.7, 52.1, 48.8

(2Z)-3-[(2,2-diethoxyethyl)carbamoyl]prop-2-enoic acid (1)

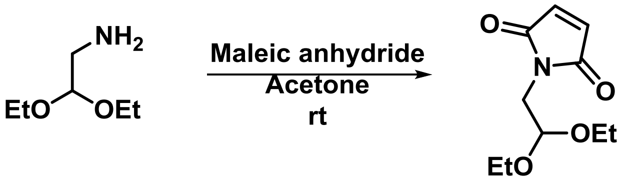

Maleic anhydride (1 eq., 10 g, 101 mmol) and 2,2-diethoxyethylamine (1 eq., 13.6 g, 14.8 mL, 101 mmol) were stirred in acetone (75 mL) at room temperature overnight. The solvent was evaporated and the residue was recrystallized from propan-2-ol. The resulting product was treated with sodium acetate (1.2 eq., 10 g, 122 mmol) in acetic anhydride (100 mL). The reaction mixture was stirred for 1h at r.t., then for 2h at 90 ^{0}C. After evaporation of acetic anhydride the residue was dissolved in EtOAc and filtered. The filtrate was concentrated *in vacuo* and the residue was purified by flash

chromatography (EtOAc/Cyclohexane gradient: 0/100 to 100/0) to give 1-(2,2-diethoxyethyl)-2,5-dihydro-1H-pyrrole-2,5-dione (15.2 g, 71.4 mmol, 70 %) as a yellow oil.

^{1}H NMR (400MHz, $CDCl_3$, δ ppm): 6.72 (s, 2 H), 4.76 (t, J=5.8 Hz, 1 H), 3.68 - 3.75 (m, 2 H), 3.67 (d, J=5.8 Hz, 2 H), 3.48 - 3.56 (m, 2 H), 1.17 ppm (t, J=7.0 Hz, 6 H)

^{13}C NMR (100MHz, $CDCl_3$, δ ppm): 170.4, 134.1, 98.4, 61.8, 39.6, 15.2

methyl 2-[(2,5-dioxo-2,5-dihydro-1H-pyrrol-1-yl)methyl]-1,3-dioxane-5-carboxylate (3)

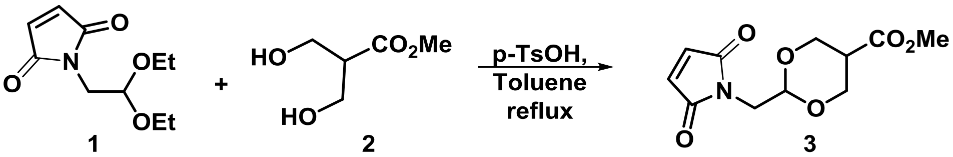

A solution of **1** (1 eq., 2 g, 9.38 mmol), **2** (1 eq., 1.26 g, 9.38 mmol) and monohydrate of *p*-toluenesulfonic acid (0.2 eq., 0.357 g, 1.88 mmol) was refluxed in toluene (100 mL) and EtOH formed during the reaction was separated as the toluene azeotrope. After 2h the toluene was evaporated, the residue was dissolved in EtOAc, washed with saturated solution of $NaHCO_3$, brine and dried over $MgSO_4$. The solvent was evaporated and the residue was purified by flash chromatography (EtOAc/Cyclohexane gradient: 0/100 to 100/0) to give methyl 2-[(2,5-dioxo-2,5-dihydro-1H-pyrrol-1-yl)methyl]-1,3-dioxane-5-carboxylate (1.96 g, 7.69 mmol, 82 %) as a mixture of cis- and trans-isomers (4:6). Individual isomers were separated for characterization.

cis-methyl-2-[(2,5-dioxo-2,5-dihydro-1H-pyrrol-1-yl)methyl]-1,3-dioxane-5-carboxylate:

^{1}H NMR (400MHz, $CDCl_3$, δ ppm): 6.72 (s, 2 H), 4.81 (t, J=5.0 Hz, 1 H), 4.58 (d, J=10.8 Hz, 2 H), 3.82 - 3.89 (m, 2 H), 3.80 (s, 3 H), 3.68 (d, J=5.3 Hz, 2 H), 2.32 (br. s., 1 H)

^{13}C NMR (100MHz, $CDCl_3$, δ ppm): 171.4, 170.3, 134.1, 97.9, 66.6, 52.3, 40.8, 39.8

MS(ESI) *m/z*: 256.07 $[M+H]^+$

trans-methyl-2-[(2,5-dioxo-2,5-dihydro-1H-pyrrol-1-yl)methyl]-1,3-dioxane-5-carboxylate:

^{1}H NMR (400MHz, $CDCl_3$, δ ppm): 6.74 (s, 2 H), 4.69 (t, J=5.1 Hz, 1 H), 4.31 (dd, J=11.8, 4.8 Hz, 2 H), 3.69 - 3.76 (m, 4 H), 3.67 (s, 3 H), 3.04 (m, 1 H)

^{13}C NMR (100MHz, $CDCl_3$, δ ppm):170.3, 170.0, 134.2, 97.5, 67.4, 51.9, 40.5, 39.7

MS(ESI) *m/z*: 256.07 $[M+H]^+$

cis-2-[(2,5-dioxo-2,5-dihydro-1H-pyrrol-1-yl)methyl]-1,3-dioxane-5-carboxylic acid (4)

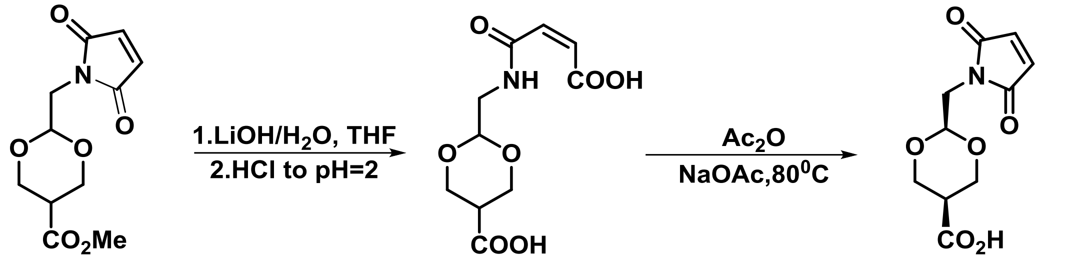

A solution of LiOH (4 eq., 0.526 g, 22 mmol) in H_2O (10 mL) was added to a solution of **3** (1 eq., 1.4 g, 5.49 mmol) in THF (20 mL) at rt. After stirring for 30min at r.t., EtOAc was added and the mixture was acidified to pH=2 with HCl. The mixture was extracted with EtOAc (3x), and the combined organic layer was washed with H_2O and brine, dried over $MgSO_4$ and concentrated to give corresponding carboxylic acid (1.38 g, 5.33 mmol, 97 %) as a mixture of cis- and trans-isomers (4:6).

The mixture (1 eq., 1.38 g, 5.33 mmol) was treated with sodium acetate (2.4 eq., 1.05 g, 12.79 mmol) in acetic anhydride (20 mL). The reaction mixture was stirred 15 min at r.t. and then for 2h at 80°C. Acetic anhydride was evaporated and water (15 mL) was added to the mixture. The mixture was stirred for 30 min and extracted with EtOAc. The organic layer was washed with brine and dried over $MgSO_4$. The solvent was evaporated and the residue was dissolved in a small amount of hot propan-2-ol and activated carbon was added. The resulting mixture was filtered off and the filtrate was kept at 0°C for 15 hours. The mixture was filtered, the precipitate was washed with cold propan-2-ol and dried to afford (2s,5s)-2-[(2,5-dioxo-2,5-dihydro-1*H*-pyrrol-1-yl)methyl]-1,3-dioxane-5-carboxylic acid (820 mg, 3.40 mmol, 62 % overall in two steps) as a white solid (t_m=178 ºC).

^{1}H NMR (400MHz, Methanol-d4, δ ppm): 6.72 (s, 2 H), 4.72 (t, J=5.1 Hz, 1 H), 4.38 (d, J=10.8 Hz, 2 H), 3.78 (dd, J=11.8, 3.3 Hz, 2 H), 3.46 (d, J=5.3 Hz, 2 H), 2.25 (br. s., 1 H).

^{13}C NMR (100MHz, Methanol-d4, δ ppm): 172.7, 170.5, 134.6, 97.2, 66.2, 40.3, 39.1

MS(ESI) *m/z*: 242.07$[M+H]^+$.

2,3,5,6-tetrafluoro-4-{[(2s,5s)-2-[(2,5-dioxo-2,5-dihydro-1H-pyrrol-1-yl)methyl]-1,3-dioxan-5-yl]carbonyloxy}benzene-1-sulfonate (5)

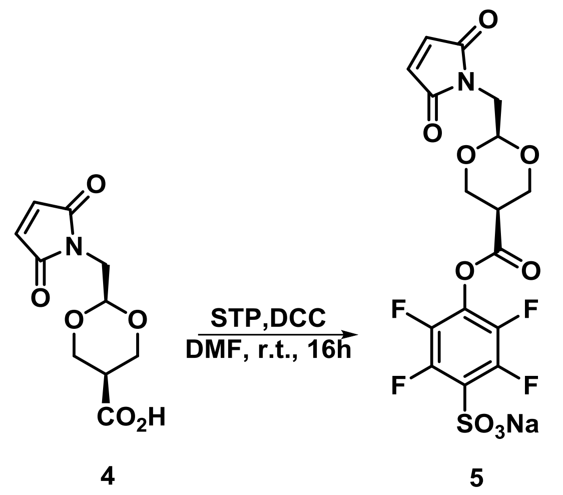

To the solution of **4** (2s,5s)-2-[(2,5-dioxo-2,5-dihydro-1H-pyrrol-1-yl)methyl]-1,3-dioxane-5-carboxylic acid (1 eq., 500 mg, 2.07 mmol) and sodium 2,3,5,6-tetrafluoro-4-hydroxybenzene-1-sulfonate (1 eq., 555 mg, 2.07 mmol) in dry DMF (5 mL) was added DCC (1.05 eq., 471 mg, 2.17 mmol) at 0 °C. The resulting mixture was stirred at 25 °C for 16h, then cooled to 0 °C, stirred for 1h and filtered off. The filtrate was diluted with cold dry Et_2O (75 mL) to precipitate the product. The mixture was filtered and the precipitate was washed with cold Et_2O and dried to give **5** (884 mg, 1.80 mmol, 87 %) as a white solid (t_m>250 ºC)

^{1}H NMR (400MHz, DMSO-d$_6$, δ ppm): 7.05 (s, 1 H), 4.86 (t, J=4.9 Hz, 1 H), 4.44 (d, J=11.5 Hz, 1 H), 4.04 (d, J=10.3 Hz, 1 H), 3.48 (d, J=4.8 Hz, 1 H), 3.18 ppm (br. s., 1 H)

^{19}F NMR (376MHz, DMSO-d$_6$, δ ppm): -139.36 (dd, J=25.18, 9.16 Hz), -153.63 (dd, J=25.18,10.31 Hz).

^{13}C NMR (126MHz, DMSO-d$_6$, δ ppm): 170.4, 168.1, 134.6, 97.4, 65.9, 40.3, 39.6.

MS(ESI) *m/z*: 468.25 [M-Na]$^-$.

Preparation of FRET probes

1. Conjugation with TAMRA-amine

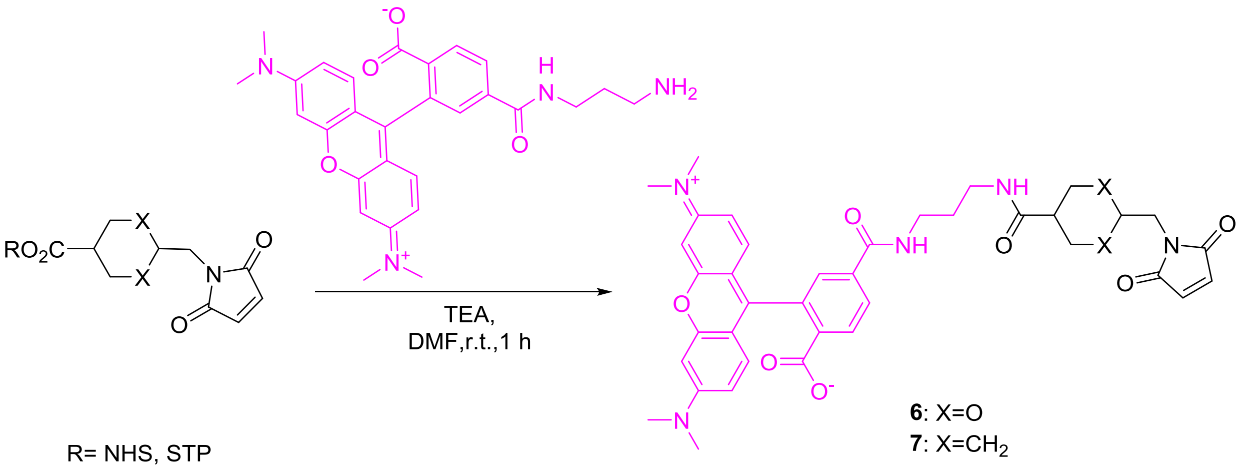

To the solution of **5** (1 eq., 0.035 mmol, 350 µL, 100 mM in DMF) was added a solution of N-(3-aminopropyl)-5-tetramethylrhodamincarboxamide (1 eq., 0.035 mmol, 350 µL, 100 mM in DMF) followed by the solution of triethylamine (3 eq., 0.105 mmol, 105 µL, 1M in DMF). The mixture was incubated at 25°C for 1 hour and purified by semi-preparative HPLC (water (0.05% TFA):acetonitrile gradient from 95:5 to 5:95 in 30 minutes) to give product **6** (22.3 mg, 0,0315 mmol, 90%) as a red solid.

^{1}H NMR (400MHz, Acetonitrile-d$_3$, δ ppm): 8.32 (d, *J*=8.3 Hz, 1 H), 8.09 - 8.18 (m, 1 H), 7.79 - 7.83 (m, 1 H), 7.75 - 7.79 (m, 1 H), 7.11 (d, *J*=9.5 Hz, 2 H), 7.06 (br. s., 1 H), 6.91 (dd, *J*=9.4, 2.4 Hz, 2 H), 6.82 (d, *J*=2.5 Hz, 2 H), 6.72 (s, 2 H), 4.76 (t, *J*=4.9 Hz, 1 H), 4.19 (d, *J*=11.8 Hz, 2 H), 3.85 - 3.94 (m, 2 H), 3.55 (d, *J*=4.8 Hz, 2 H), 3.41 (q, *J*=6.1 Hz, 2 H), 3.30 (q, *J*=6.3 Hz, 2 H), 3.21 - 3.26 (m, 12 H), 2.16 (br. s., 1 H), 1.68 - 1.76 (m, 2 H)

HR-ESI-MS: $C_{38}H_{39}N_5O_9$, 709.27478; found 709.27593

For the preparation of MCC-TAMRA conjugate **7** the same protocol was used with the commercial sulfo-SMCC instead of **5** to give **7** (22.9 mg, 0,0325 mmol, 93%) as a red solid.

^{1}H NMR (400MHz, Methanol-d4, δ ppm): 8.69 (m, 1H), 8.33 (m, 1H), 8.14 (m, 1H), 7.71-7.87 (m, 2 H), 7.08 (d, *J*=8.8 Hz, 2 H), 6.94 (d, *J*=9.5 Hz, 2 H), 6.87 (d, *J*=1.8 Hz, 2 H), 6.70 (s, 2 H), 3.31 (t, *J*=6.5 Hz, 2 H), 3.22-3.25 (m, 2H), 3.20 (s, 12 H), 3.13 (t, *J*=6.7 Hz, 2 H), 1.93 - 2.05 (m, 1 H), 1.62 - 1.75 (m, 4 H), 1.58 (d, *J*=13.3 Hz, 2 H), 1.46 - 1.54 (m, 1 H), 1.15 - 1.34 (m, 2 H), 0.80 - 0.95 (m, 2 H)

HR-ESI-MS: $C_{40}H_{43}N_5O_7$, 705.31625; found 705.31785

2. Conjugation with BHQ-2-thiol

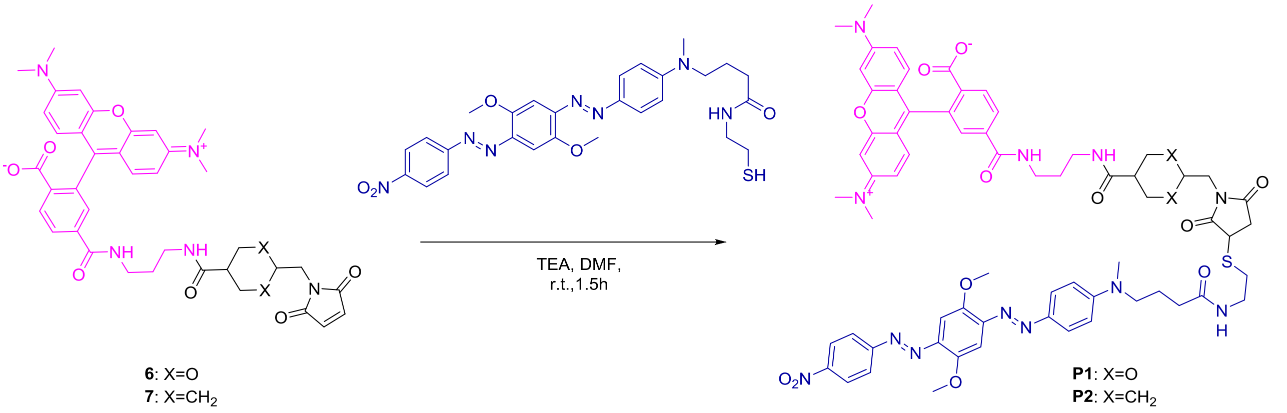

To the solution of **6** (1 eq., 0.015 mmol, 300 µL, 50 mM in DMF) was added a solution of 4-((4-((E)-(2,5-dimethoxy-4-((E)-(4-nitrophenyl)diazenyl)phenyl)diazenyl)phenyl)(methyl)amino)-N-(2-mercaptoethyl)butanamide (BHQ-2-SH) (1 eq., 0.015 mmol, 300 µL, 50 mM in DMF) followed by the solution of triethylamine (3 eq., 0.045mmol, 45 µL, 1M in DMF). The mixture was incubated at 25°C for 1.5 hour and purified by semi-preparative HPLC (water (0.05% TFA): acetonitrile gradient from 95:5 to 5:95 in 30 minutes) to give product **P1** (16.2 mg, 0,0128 mmol, 85%) as a violet solid.

^{1}H NMR (400MHz, DMSO-d$_6$, δ ppm): 8.81 (t, *J*=5.3 Hz, 1 H), 8.43 (d, *J*=8.8 Hz, 2 H), 8.29 - 8.33 (m, 1 H), 8.24 - 8.29 (m, 1 H), 8.00 - 8.12 (m, 3 H), 7.95 (s, 1 H), 7.80 (d, *J*=9.0 Hz, 2 H), 7.72 (t, *J*=5.4 Hz, 1 H), 7.42 (s, 1 H), 7.36 (s, 1 H), 7.03 (s, 4 H), 6.91 (s, 2 H), 6.87 (d, *J*=9.0 Hz, 2 H), 4.75 (t, *J*=5.1 Hz, 1 H), 4.29 (d, *J*=11.5 Hz, 2 H), 4.02 - 4.07 (m, 1 H), 3.99 (s, 3 H), 3.94 (s, 3 H), 3.81-3.84 (m, 2H), 3.44 - 3.51 (m, 4 H), 3.30 - 3.36 (m, 4 H), 3.24 (s, 12H), 3.20 (d, *J*=8.3 Hz, 2 H), 3.07 (s, 3 H), 2.84 (dt, *J*=13.0, 6.4 Hz, 1 H), 2.71 (dt, *J*=13.2, 6.7 Hz, 1 H), 2.55-2.57 (m, 2H), 2.34 (br.s, 1H), 2.18 (d, *J*=5.5 Hz, 2 H), 1.76 - 1.86 (m, 2 H), 1.66 - 1.76 (m, 2 H)

HR-ESI-MS: $C_{65}H_{70}N_{12}O_{14}S$, 1274.48552; found 1274.48491.

For the preparation of conjugate **P2** the same protocol was used with **7** to give **P2** (15.4 mg, 0,0122 mmol, 81%) as a violet solid.

1H NMR (400MHz, DMSO-d6, δ ppm): 8.74 (t, *J*=5.1 Hz, 1 H), 8.42 (d, *J*=8.8 Hz, 2 H), 8.29 (d, *J*=8.3 Hz, 1 H), 8.23 (d, *J*=7.5 Hz, 1 H), 8.04 (d, *J*=8.8 Hz, 3 H), 7.92 (s, 1 H), 7.81 (d, *J*=8.8 Hz, 2 H), 7.71 (t, *J*=5.1 Hz, 1 H), 7.42 (s, 1 H), 7.35 (s, 1 H), 7.01 (br. s., 4 H), 6.84 - 6.93 (m, 4 H), 4.01 - 4.06 (m, 1 H), 3.99 (s, 3 H), 3.93 (s, 3 H), 3.47 (t, *J*=6.3 Hz, 1 H), 3.17-3.32 (m, 18 H), 3.03-3.10 (m, 5 H), 2.70 - 2.93 (m, 2 H), 2.55-2.57(m, 2H), 2.13 - 2.22 (m, 2 H), 1.81 (br. s., 2 H), 1.58-1.72 (m, 6H), 1.20 - 1.28 (m, 4 H), 0.82 - 0.96 (m, 2 H)

HR-ESI-MS: $C_{67}H_{74}N_{12}O_{12}S$, 1270.52699; found 1270.52364

Stability of MD-based *vs* MCC-based probes in human plasma and other media

General procedure

Aliquots of stock solutions of probes **P1** and **P2** (10 mM in DMSO, stored at -80 ˚C) were diluted with DMSO to give 40 μM working solutions. 25 μL of each working solution was added to 975 μL of human plasma or other media, vortexed and distributed onto 96-well plates (in triplicates). The fluorescence was measured every 3 minutes for 15 hours and normalized according to the fluorescence of a solution of TAMRA-NH_2 (1 μM) and BHQ-2-SH (1 μM) in appropriate media (positive control). Obtained results are shown in Table S1. Normalized fluorescence of probes **P1** and **P2** after 12h and 72h in different media is shown in Table S2.

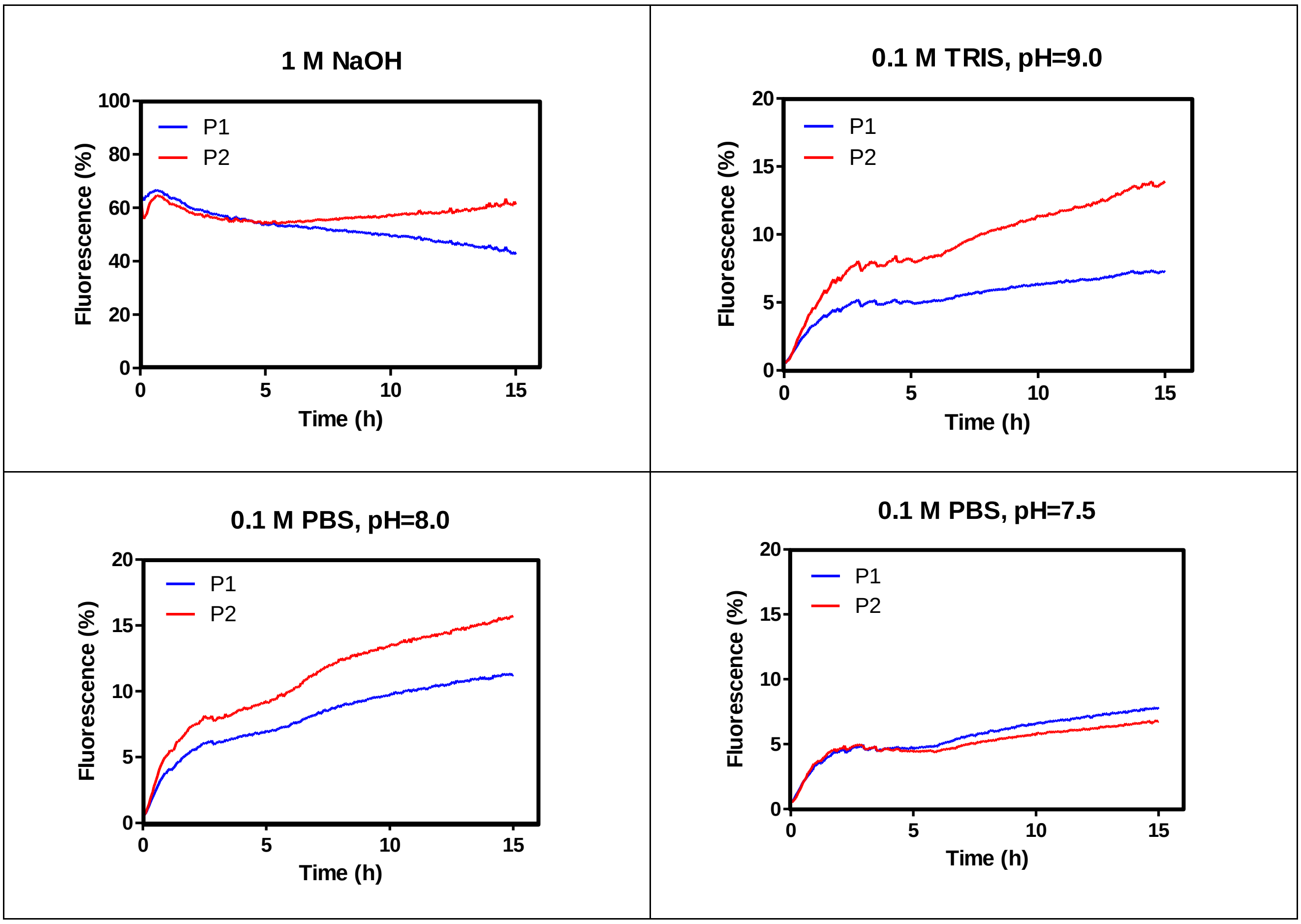

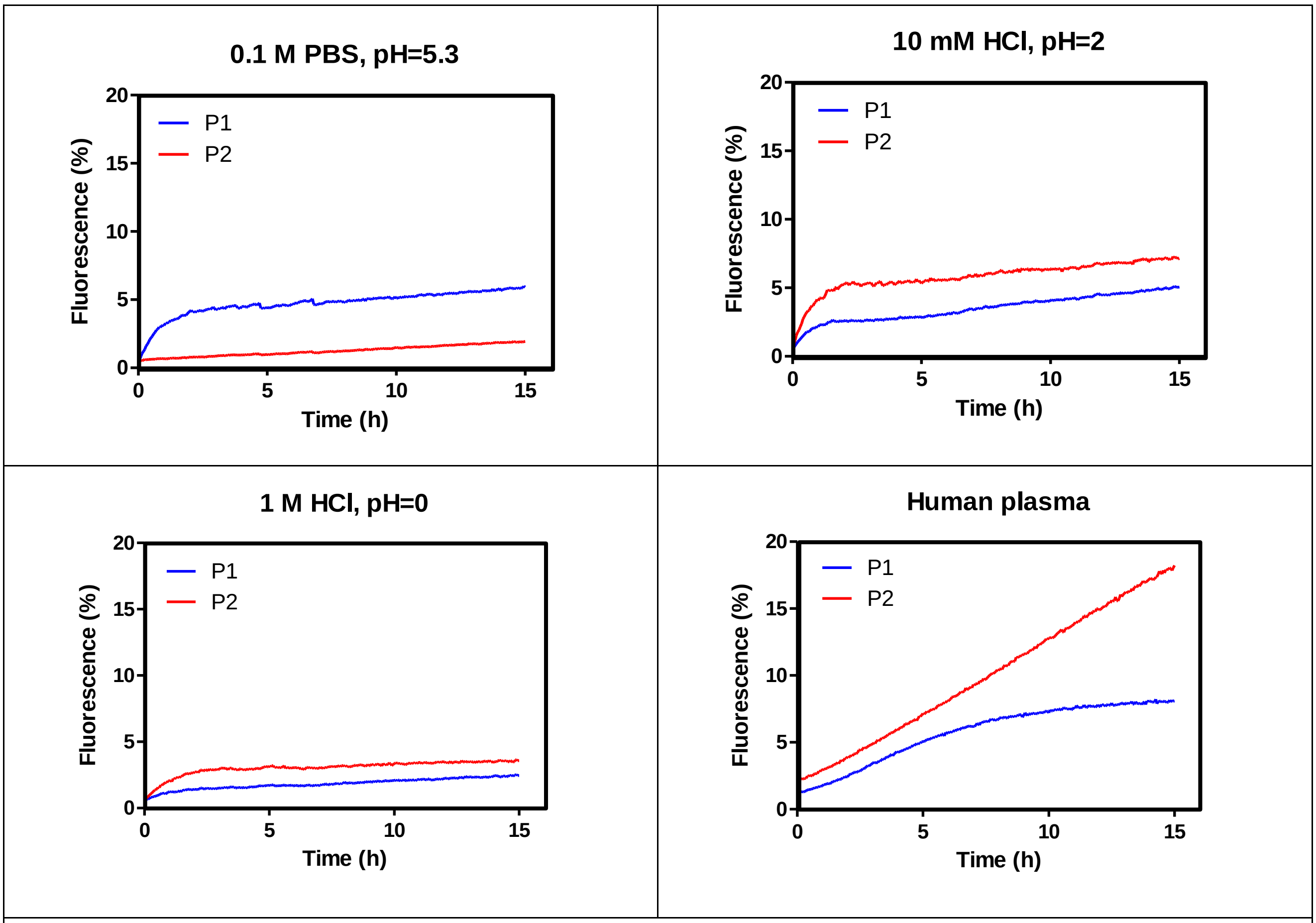

Table S1. Evolution in time of the fluorescence of MD-based (**P1**) and MCC-based (**P2**) probes (1µM) in different media at 37 °C. Excitation at 550 nm, emission at 580 nm.

Condition	Fluorescence %			
	**P1**		**P2**	
	12h	72h	12h	72h
Plasma, pH=7.8	8%	8%	**15%**	**40%**
TRIS 0.1M, pH=9.0	7%	8%	12%	**16%**
PBS 10mM, pH=7.5	7%	9%	6%	9%
10mM HCl, pH=2.0	5%	8%	7%	10%
1M HCl, pH=0	2%	5%	4%	5%

Table S2.Normalized fluorescence of MD-based (**P1**) and MCC-based (**P2**) probes (1µM) after 12h and 72h in different media at 37 °C.

Rate of succinimide hydrolysis of probe P1 in human plasma

P1 and **P2** FRET probes at 50µM (final concentration) were incubated in 2 mL of human plasma (DMSO 10%) at 37 ˚C. After defined intervals of time 100µL aliquots were mixed with 100µL of acetonitrile, allowing the precipitation of proteins, the resulting mixture was centrifuged and the

supernatant was analysed by HPLC. The conversion was calculated as the area under hydrolyzed product (**hP1**) divided by the total area.

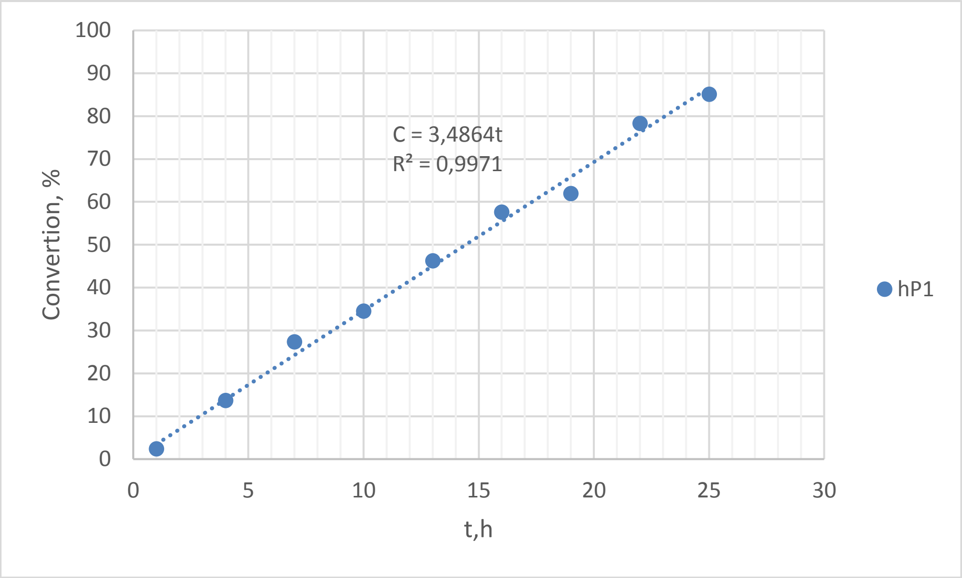

Preparation of antibody conjugates with DAR 8

1. Reduction of the antibody

Trastuzumab was dissolved in 10 mM PBS (pH 7.5) containing EDTA (2mM) to give 5 mg/mL solution. To this solution was added a solution of TCEP (4.8 eq., 100 mM in H2O). The mixture was incubated at 37°C for 2 h and used in the next without further purification.

2. Conjugation with cysteines

To 100 µL of the solution of the reduced trastuzumab was added the solution of **6** (30 eq., 4.13µL, 25 mM in DMF) or **7** (20 eq., 2.75µL, 25 mM in DMF) at 0 °C. The resulting mixture was incubated at 4 °C for 1h and then purified using Bio-Spin P-30 Columns (Bio-Rad, Hercules, U.S.A.) equilibrated with 10 mM PBS (pH 7.5). Conjugates were analyzed mass spectrometry.

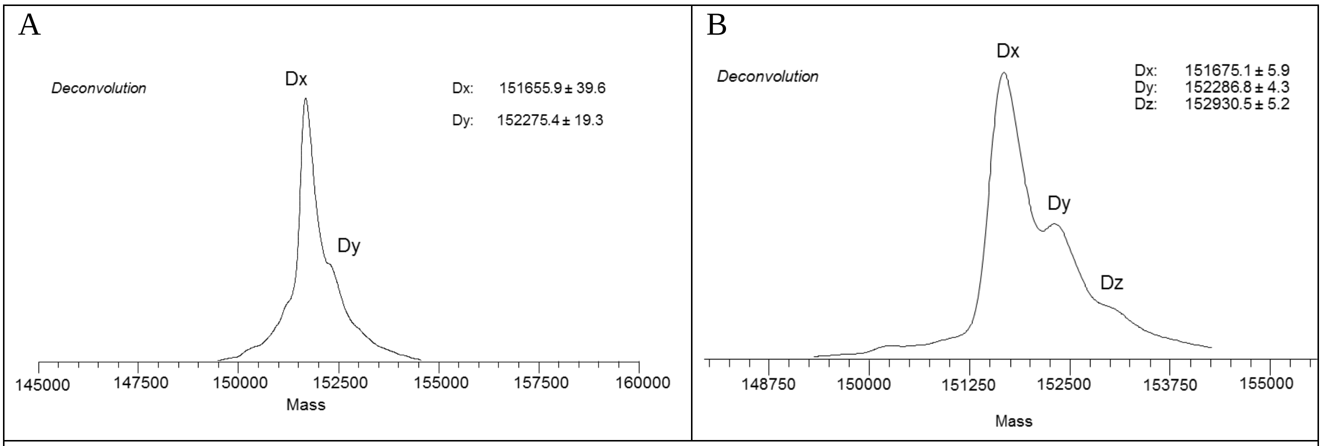

Figure S. Deconvoluted mass spectra of the deglycosylated antibody conjugates. A. Conjugate with Dye-to-Antibody ratio (DAR) = 8.1 prepared using MD-TAMRA. B. Conjugate prepared using MCC-TAMRA with DAR = 8.3.

Characterization of conjugates

Prior to native MS experiments, antibody-dye conjugates (ADCs) were desalted against 150 mM ammonium acetate solution buffered at pH 7.4 using six cycles of concentration/dilution on micro-concentrators (Vivaspin, 30 kD cutoff, Sartorius, Gottingen, Germany). Protein concentration was determined by UV absorbance using a NanoDrop spectrophotometer (Thermo Fisher Scientific, Illkirch, France). ADC deglycosylation was achieved by incubating (37 °C – 2h) 0.4 units of Remove-iT® Endo S (New England Biolabs, Ipswich, U.S.A.) per microgram of ADC prior to buffer exchange desalting step.

MS experiments were performed on an electrospray time-of-flight mass spectrometer MS (LCT, Waters, Manchester) coupled to an automated chip-based nanoelectrospray device (Triversa Nanomate, Advion Biosciences, Ithaca, U.S.A.) operating in the positive ion mode. For native MS experiments, external calibration of the ESI-TOF instrument was performed using singly charged

ions produced by a 2 mg/mL solution of cesium iodide in 2-propanol/water (1v/1v). Tuning parameters of the mass spectrometer were carefully optimized to improve desolvation and ion transfer as well as maintaining weak interactions. Particularly, the sample cone voltage Vc was set to 120 V and the backing pressure Pi was increased to 6 mbar to improve ion collisional cooling and maintain non-covalent interaction for averaging DAR calculation. Native MS data interpretation was performed using MassLynx 4.1 (Waters, Manchester, UK.).

Average DAR values from native MS were calculated from the relative peak intensities measured from the raw mass spectrum (taking into account 21^+ to 26^+ charge states). Average DAR value was obtained by summing up the weighted peak percentage from all observed species and dividing the sum by 100, as follows: DAR = Σ(relative peak intensity × number of loaded dye)/100 (number of dye load).

Stability of antibody-dye conjugates in human plasma

10µL of MD-based and MCC-based antibody dye conjugates (5mg/mL) were maintained in PBS buffer (10 mM, pH = 7,54) at 37 ˚C for 70h to achieve hydrolysis of succinimide ring, then diluted with 40uL of milli-Q water and 50µL of human plasma and incubated at 37 ˚C. Every 24h aliquots (10µL) were taken and stored at -20 ˚C. To perform SDS-PAGE analysis of the resulted samples the aliquots were diluted with 50 volumes of water in order to decrease a concentration of proteins in the mixture to 1 mg/mL. Reducing glycine-SDS-PAGE was performed on 4–15% Mini-PROTEAN® TGX™ Gel (Bio-Rad ref 4561084) following standard lab procedures. Samples (25 µL) were mixed with 25 µL of loading buffer (2x Laemmli Sample Buffer, Bio-Rad ref 1610737) containing 5% v/v of β-mercaptoethanol and heated at 95 °C for 5 minutes. The gel was run at constant voltage (200 V) for 40 min using TRIS 0.25 M - Glycine 1.92 M - SDS 1% as a running buffer. Fluorescence was visualized on GeneGenius bio-imaging system (Syngene) prior to staining with Coomassie Blue.

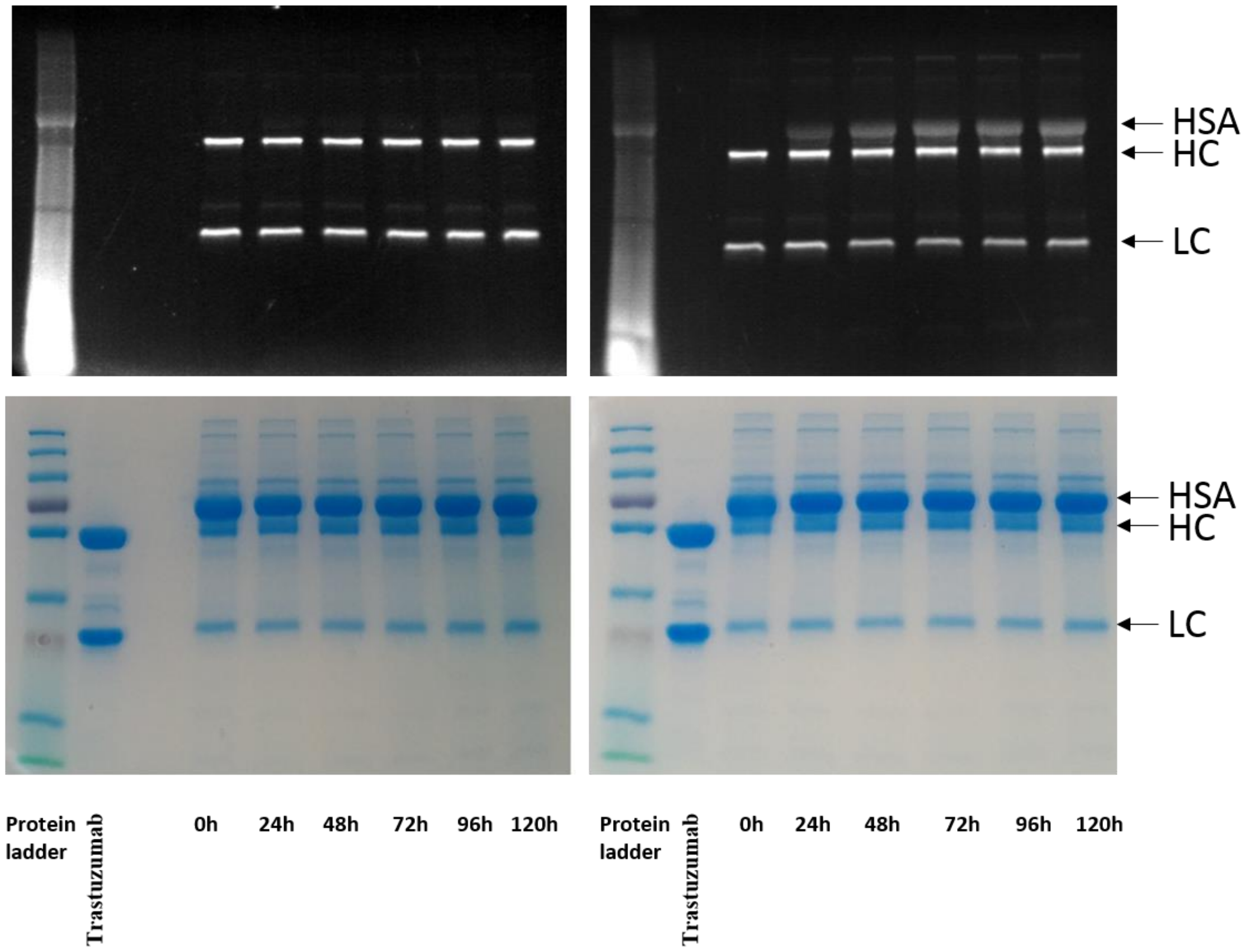

HSA
HC
LC
HSA
HC
LC
Protein ladder
Trastuzumab
0h
24h
48h
72h
96h
120h
Protein ladder
Trastuzumab
0h
24h
48h
72h
96h
120h
